# Literature survey on epidemiology and pathology of cardiac fibroma

**DOI:** 10.1186/2047-783X-17-5

**Published:** 2012-03-27

**Authors:** Suguru Torimitsu, Tetsuo Nemoto, Megumi Wakayama, Yoichiro Okubo, Tomoyuki Yokose, Kanako Kitahara, Tsukasa Ozawa, Haruo Nakayama, Minoru Shinozaki, Daisuke Sasai, Takao Ishiwatari, Kensuke Takuma, Kazutoshi Shibuya

**Affiliations:** 1 Department of Surgical Pathology, Toho University School of Medicine, 6-11-1 Omori-Nishi, Ota-Ku, Tokyo 143-8541, Japan; 2 Department of Pathology, Kanagawa Cancer Center, 1-1-2, Nakao, Asahi-Ku, Yokohama-city, Kanagawa 245-0815, Japan; 3 Department of Internal Medicine, Division of Rheumatology, Toho University School of Medicine, 6-11-1 Omori-Nishi, Ota-Ku, Tokyo 143-8541, Japan; 4 Division of Cardiovascular Surgery, Department of Surgery (Omori), Toho University School of Medicine, 6-11-1 Omori-Nishi, Ota-Ku, Tokyo 143-8541, Japan; 5 Department of Neurosurgery, Toho University Ohashi Medical Center, 2-17-6, Ohashi, Meguro, Tokyo 153-8515, Japan; 6 Department of Gastroenterology and Hepatology, Omori Medical Center, Toho University School of Medicine, 6-11-1 Omori-Nishi, Ota-Ku, Tokyo 143-8541, Japan; 7 Department of Dermatology, Peking University First Hospital, Beijing, China

## Abstract

**Background:**

Although cardiac fibroma has been regarded as benign tumor, it presents various symptoms and may lead to death. Unfortunately, only a few studies have reported the epidemiology, embryology, and histopathology of the tumor, and the factors predicting poorer outcome are still obscured.

**Methods:**

In July 2011 we searched for English and Japanese cases of cardiac fibroma using the PubMed and IgakuChuoZasshi databases. We then extracted and sampled raw data from the selected publications in accordance with the Preferred Reporting Items for Systematic Reviews and Meta-Analyses (PRISMA) style as much as was possible.

**Results:**

Details of a total of 178 patients with cardiac fibroma were retrieved. The mean age was 11.4 years (median: 2.8 years). Tumor sizes ranged from 8.0 to 150.0 mm (mean 53.1 mm). The left ventricle was found to be the most common site associated with the tumor at a rate of 57.3%, followed by the right ventricle, and interventricular septum. The highest mortality was found in patients with septal involvement (58.6%). In all, 111 patients survived among the 160 patients with a recorded outcome. A younger age of the patient at the time of diagnosis was associated with a decreased survival rate. In addition, a significant positive association was found between ages for patients younger than 17 years of age and the diameter of the tumor at the time of diagnosis (r = 0.341, *P *= 0.006).

**Conclusions:**

Both the younger age of patients at the time of diagnosis and septal involvement can be regarded as factors significantly indicating a poor prognosis. Furthermore, our statistical analyses support the following hypotheses. First, the high ratio of tumor-to-heart size may generate low cardiac output and therefore lead to poor outcome. Second, the ratio of the sites where cardiac fibroma occurred corresponds with the ratio of the muscular weight of the cardiac chamber. Third, cardiac fibroma involving the interventricular septum more frequently induces conduction system disease.

## Background

Primary cardiac tumors are extremely rare, with a reported incidence ranging from 0.0017 to 0.019% [1]. Of such tumors, about 75% are benign nature [2,3]. Cardiac fibroma is the second most common primary cardiac tumor in infants and young children after rhabdomyoma [4,5]. Although cardiac fibroma is a benign and solitary tumor [6] composed of fibroblasts and collagen [7,8], it is clinically important as it may present with symptoms such as inflow and outflow obstruction, conduction system disease, and sudden death [9,10]. In contrast to cardiac rhabdomyoma, cardiac fibroma rarely regresses spontaneously [11] and surgical removal in a symptomatic case should be considered [12,13]. Unfortunately, only a few studies have reported the epidemiology, embryology, and histopathology of the cardiac fibroma. Thus, we searched literature databases in July 2011 and selected publications related to cardiac fibroma using a modification of our previously reported method [14] to elucidate the factors predicting poor outcome.

## Methods

### Data collection

In July 2011 we searched English and Japanese languages case reports of cardiac fibroma using PubMed http://www.ncbi.nlm.nih.gov/pubmed/ and IgakuChuoZasshi http://www.jamas.or.jp/ databases by conducting a search for 'fibroma' with no time limitations. In the PubMed search, 'case report' and 'English' options were used as an additional tool, and the PubMed system automatically conducted 'Medical Subject Headings' or 'All Fields' searches for 'fibroma'.

In contrast, for the IgakuChuoZasshi search the 'shoreihoukoku' (Japanese word meaning case report) option was used as an additional tool. We then reviewed the searched abstracts of publications selected from the databases to determine the cumulative number of reported cardiac fibroma cases. Additionally, we reviewed the references of the selected publications in order to find and include publications that were not recorded in the databases.

We then extracted and sampled the raw data from the selected publications and our autopsy case in relation to factors such as age, gender, clinical symptoms, surgical intervention, outcome, site of the tumor, and maximum diameter of the tumor. In the present study, data collection was performed in accordance with the Preferred Reporting Items for Systematic Reviews and Meta-Analyses (PRISMA) style [15] as much as was possible.

### Statistical analyses

#### Comparison of survival

To clarify poor prognostic factors in patients with cardiac fibroma, we compared the patient age and maximum diameter of the tumor at the time of diagnosis between patients that survived and those that did not survive. The analyses were performed using the Mann-Whitney U test.

#### Comparison of gender

To determine whether there were gender differences in patients with cardiac fibroma, we compared the patient age and maximum diameter of the tumor at the time of diagnosis between males and females with cardiac fibroma. The analyses were performed using the Mann-Whitney U test.

#### Correlation between patient age and tumor size

We used the Spearman correlation coefficient to know the correlation between the patient age and maximum diameter of the tumor at the time of diagnosis. Since it has been reported [16] that the growth of the heart is usually completed at 17 to 20 years of age, we also calculated the Spearman correlation coefficient between the patient age and maximum diameter of the tumor at the time of diagnosis in patient groups under 17 years of age and those over 17 years of age, independently.

#### Relative size of tumor of cardiac fibroma

Since younger individuals have smaller hearts than older individuals [17], analyses using mere tumor size may be insufficient to assess the effect of tumor size on survival, and a consideration of physiological cardiac growth should be required. Therefore, we calculated the tumor-to-heart size ratio of patients by dividing the maximum diameter of the tumor (DT) by the physiological cardiac weight (CW) [17], and created a scatter plot of DT/CW. We then used the Spearman rank correlation coefficient to confirm the correlation between DT/CW and the patient age at the time of diagnosis. Additionally, to confirm the correlation between DT/CW and patient outcome, we used the Mann-Whitney U test to compare DT/CW between patients that survived and those that did not survive.

#### Correlation between tumor occurrence and cardiac chamber weight

To confirm the correlation between tumor occurrence and cardiac chamber weight, we compared the ratio of the number of tumor sites and the muscular weight of the cardiac chamber using the χ^2 ^test. Muscular weight was determined using examples of previous publication reported by Sandstede *et al. *[18]. To determine the weight of the interventricular septum in the present study, we divided the left ventricle into four zones comprising anterior wall, lateral wall, posterior wall, and interventricular septum.

#### Correlation between site of cardiac fibroma and outcome

Since a previous report indicated that patients with interventricular septal cardiac fibroma have a poorer outcome [19], we used the χ^2 ^test to compare mortality between patients whose condition involving or not involving the interventricular septum. We then compared DT/CW between these groups of patients using a Mann-Whitney U test, and compared the incidence of arrhythmia between these groups of patients using the χ^2 ^test.

Differences were considered significant at *P *< 0.05 in all statistical analyses.

## Results

### Data collection

We retrieved 4,787 English and 1,901 Japanese (total 6,688) publications by conducting a search for 'fibroma' using PubMed and IgakuChuoZasshi databases. Of these publications, 99 English and 6 Japanese manuscripts were selected as describing representative cases of cardiac fibroma, with the remaining 6,583 publications being excluded. We then reviewed the references of these 105 selected publications and included another 16 Japanese publications. These 121 publications contained reports concerning 177 patients with cardiac fibroma. We added our autopsy patient to this list and thus extracted data from a total of 178 patients.

The patient age at the time of diagnosis ranged from 0 (including prenatal cases) to 83 years (n = 160, 18 not reported, mean ± SD: 11.4 ± 18.5) and the median age at the time of diagnosis was 2.8 years. A total of 65 patients, or 40.6% of the total, were in the group comprising patients less than 1 year of age, and 18 patients, or 11.3% of the total, were in the group comprising prebirth or neonatal cases. The gender ratio was 92:78 male to female (n = 170, gender not reported in 8 cases). The maximum diameter of the tumor at the time of diagnosis ranged from 8.0 to 140.0 mm (n = 92, 86 not reported, mean ± SD: 52.9 ± 23.9 mm). The left ventricle (98/171, 57.3%) was found as the commonest site of the tumor, followed by the right ventricle (47/171, 27.5%), interventricular septum (29/171, 17.0%), right atrium (9/171, 5.3%), and left atrium (3/171, 1.8%). In all, 18 patients had cardiac fibroma that involved 2 or 3 continuous sites of the myocardium. The outcome of 160 patients was described: 111 patients survived and 49 died. Surgical intervention for the cardiac fibroma was undertaken in 119 of the 178 cases, of which 87 patients survived and 22 died (10 not reported).

### Statistical analysis

#### Comparison of survival

We confirmed that 111 patients with cardiac fibroma survived and 49 patients died.

Of these 160 patients, the age of 142 patients was documented. The mean age ± SD of the group that survived was 13.5 ± 18.4 years (n = 93), while for the group that did not survive it was 5.8 ± 16.2 years (n = 49). The group that did not survive was significantly younger than the group that survived (Mann-Whitney U test: *P *< 0.001). Of the 160 cases, 82 included reports of the maximum diameter of the tumor at the time of diagnosis. The mean ± SD tumor diameter of the group that survived was 53.3 ± 25.0 mm (n = 62), while that of the group that did not survive was 48.7 ± 19.4 mm (n = 20). No significant difference was found between these groups in regard to tumor diameter (Mann-Whitney U test: *P *= 0.810).

These data are summarized in Table 1

**Table 1:** Differences between gender and patient outcome

	Male	Female	*P *value	Survival	Dead	*P *value
Age, years	10.8 ± 18.4 (n = 84)	12.2 ± 18.6 (n = 68)	0.529	13.5 ± 18.4 (n = 93)	5.8 ± 16.2 (n = 49)	< 0.001
Diameter, mm	51.6 ± 23.5 (n = 49)	54.7 ± 24.1 (n = 43)	0.562	53.3 ± 25.0 (n = 62)	48.7 ± 19.4 (n = 20)	0.810

Age is age at time of diagnosis, diameter is the maximum diameter at time of diagnosis. Values are mean ± SD, *P *values by Mann-Whitney test.

#### Comparison of gender

The mean ± SD age of males and females at the time of diagnosis was 10.8 ± 18.4 years (n = 84) and 12.2 ± 18.6 years (n = 68), respectively (gender not reported for 26 patients). No significant difference was found for patient age between these groups (Mann-Whitney U test: *P *= 0.529). The mean ± SD tumor diameter of males and females was 51.6 ± 23.5 mm (n = 49) and 54.7 ± 24.1 mm (n = 43), respectively (size not reported for 85 patients). No significant difference was found in tumor diameter between these groups (Mann-Whitney U test: *P *= 0.562). These data are summarized in Table 1. A total of 23 of the 84 (27.4%) male patients and 20 of the 67 (29.9%) female patients died (outcome not reported for 27 patients). No significant difference was found in mortality between these groups (χ^2^, *P *= 0.738).

#### Correlation between patient age and tumor size

Our literature survey revealed that 18 of the 178 patients with cardiac fibroma (11.3%) were diagnosed during the prenatal or neonatal period, and that this tumor had a certain size regardless of patient age. Calculation of the Spearman correlation coefficient using the patient age and maximum diameter of the tumor at the time of diagnosis (n = 92, 85 not reported) showed a significant positive association between these factors (r = 0.338, *P *= 0.001).

The Spearman correlation coefficient was calculated between ages for patients under 17 years of age (n = 64) and for those over 17 years of age (n = 28) and tumor diameter. Results showed a significant positive correlation between patient age and tumor diameter (r = 0.341, *P *= 0.006) for patients under 17 years of age, while there was no significant correlation between these factors for patients over 17 years of age (r = -0.080, *P *= 0.676).

#### Relative size of tumor of cardiac fibroma

**Figure 1:**
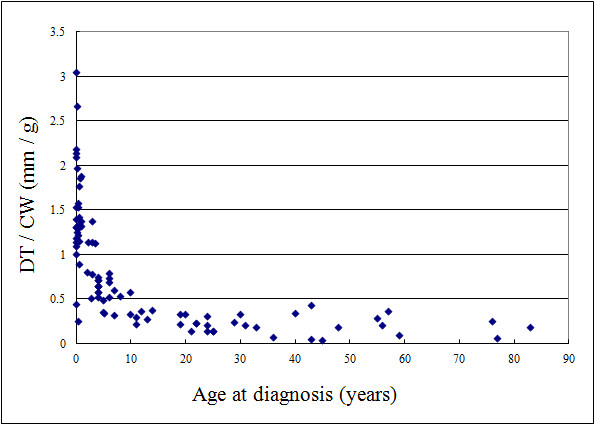
**Scatter plot of diameter of the tumor/cardiac weight (DT/CW) and the patient age at the time of diagnosis**. DT/CW was calculated by dividing the diameter of the tumor by the physiological cardiac weight. Analysis of these factors using Spearman's rank correlation coefficient showed a significant negative association (r = -0.863, *P *< 0.001).

The scatter plot of DT/CW and patient age at the time of diagnosis (Figure 1) showed a significant negative association (Spearman's rank correlation coefficient, r = -0.863, *P *< 0.001). Furthermore, a significant difference was found in DT/CW between patients that survived (n = 62, mean ± SD: 0.67 ± 0.57 mm/g) and those that did not survive (n = 20, mean ± SD: 1.16 ± 0.75 mm/g) (Mann-Whitney U test: *P *= 0.008).

#### Correlation between site of tumor occurrence and cardiac chamber weight

The present study found that the left ventricle was the most common site associated with cardiac fibroma (98/171, 57.3%), followed by the right ventricle (47/171, 27.5%) and interventricular septum (29/171, 17.0%). We then investigated the correlation between the site of tumor occurrence and cardiac chamber weight, and no significant difference was found in ratio between the number of tumor sites (left ventricle: right ventricle: interventricular septum, 98:47:29) and muscular weight of the cardiac chamber (left ventricle free wall: right ventricle: interventricular septum, 99:45:33) (χ^2 ^test, *P *= 0.869).

#### Correlation between site of cardiac fibroma and outcome

The number of patient deaths involving the interventricular septum as the primary site of cardiac fibroma (17/29, 58.6%) was significantly higher than that involving other sites (30/126, 23.8%) (χ^2 ^test, *P *< 0.001). No significant difference was found in DT/CW between patients associate with interventricular septal involvement of cardiac fibroma (n = 18, mean ± SD: 1.00 ± 0.78 mm/g) and other sites (n = 89, mean ± SD: 0.91 ± 0.79 mm/g) (Mann-Whitney U test, *P *= 0.917).

The number of patients showing arrhythmia with cardiac fibroma involving the interventricular septum and other sites was 16/29 (55.2%) and 40/126 (31.7%), respectively. The former ratio is significantly higher than the latter ratio (χ^2 ^test, *P *= 0.018).

## Discussion

To elucidate the significant poor prognostic factors associated with the cardiac fibroma, we collected data in accordance with PRISMA style as much as was possible. Here, we wish to discuss the findings that emerged from our survey and a specific case we encountered in relation to cardiac fibroma.

In the present study, patients that did not survive were significantly younger than those that did. This suggests that a younger age at the time of diagnosis of cardiac fibroma is associated with a poorer outcome. However, no significant difference was found in the maximum diameter of the tumor between both groups. Given that younger individuals have a smaller heart than older individuals [17], the high ratio of tumor-to-heart size may generate low cardiac output and therefore lead to a poorer outcome. To confirm our hypothesis, we appraised the tumor-to-heart size ratio of the patients by dividing DT by the physiological CW [17]. The scatter plot of DT/CW and the patient age at the time of diagnosis (Figure 1) showed a significant negative association (Spearman's rank correlation coefficient, r = -0.863, *P *< 0.001). Furthermore, a significant difference was found in DT/CW between patients that survived (n = 62, mean ± SD: 0.67 ± 0.57 mm/g) and those that did not survive (n = 20, mean ± SD: 1.16 ± 0.75 mm/g) (Mann-Whitney U test: *P *= 0.008). These findings suggest that a high DT/CW indicating largeness in relative size of tumor is a poor prognostic factor for cardiac fibroma.

Our literature survey also revealed that 18 of 178 patients with cardiac fibroma (11.3%) were diagnosed during the prenatal or neonatal period, and that this tumor had a certain size regardless of the patient's age. These facts suggest that cardiac fibroma may be a congenital disorder. Additionally, we calculated the Spearman correlation coefficient to know the correlation between the patient age and tumor diameter at the time of diagnosis (n = 92, 85 not reported). The results showed a significant positive association between them (r = 0.338, *P *< 0.001), indicating that the tumor grew with age. However, we described previously that no significant difference was found in tumor diameter between the (older) group that survived and the (younger) group that did not survive. To better understanding the apparent paradoxical results of our statistical analyses, we suggested the hypothesis that this tumor stops growing at a certain age. According to a previous publication [16], the growth of the heart is usually completed at 17 to 20 years of age. Therefore, we calculated the Spearman correlation coefficient between the patient age in patients under 17 years of age (n = 64) and in patients over 17 years of age (n = 28) tumor diameter. The former group showed significant positive correlation between the patient age and tumor diameter (r = 0.341, *P *= 0.006), while the latter group showed no significant correlation (r = -0.080, *P *= 0.676) between these factors. These results suggest that the tumor grew with physiological cardiac growth.

The present study revealed that the left ventricle was the most common site associated with cardiac fibroma (98/171, 57.3%), consistent with previous reports [20,21], followed by the right ventricle (47/171, 27.5%) and the interventricular septum (29/171, 17.0%).

We then investigated the correlation between the ratio of the number of tumor sites (left ventricle: right ventricle: interventricular septum, 98:47:29) and the cardiac chamber weight (left ventricle free wall: right ventricle: interventricular septum, 99:45:33) using the χ^2 ^test. No significant difference was found in ratio between the number of tumor sites and the cardiac chamber weight (*P *= 0.869). This finding indicated that the ratio of the site where cardiac fibroma occurred corresponded with the ratio of the muscular weight of the cardiac chamber.

The present study also shed some light on the correlation between the site of cardiac fibroma and outcome. The number of patient deaths involving the interventricular septum as the primary site of cardiac fibroma (17/29, 58.6%) was significantly higher in comparison to deaths not involving the interventricular septum (30/126, 23.8%) (χ^2 ^test, *P *< 0.001). However, no significant difference was found in DT/CW between patients associated with interventricular septal involvement (n = 18, mean ± SD: 1.00 ± 0.78 mm/g) and those associated with other sites (n = 89, mean ± SD: 0.91 ± 0.79 mm/g) (Mann-Whitney U test, *P *= 0.917). These results suggested that septal involvement is a significant prognostic factor for the tumor, regardless of the diameter relative to heart size. To account for this phenomenon, the tumor involving the interventricular septum may induce malfunctioning in conduction system. We therefore compared the prevalence of arrhythmia in patients with the tumor involving between septum and other sites. The group with septal involvement comprised 16 of 29 (55.2%) patients, while another group comprised 40 of 126 (31.7%) patients. The former ratio is significantly higher than the latter's (χ^2 ^test, *P *= 0.018). Hence there is a tendency for cardiac fibroma located at the interventricular septum to cause arrhythmias (Table 2).

**Table 2:** Differences between interventricular (IVS) septum and other cardiac sites

	IVS	Not IVS	*P *value
Mortality, %	58.6 (17/29)	23.8 (30/126)	< 0.001^a^
DT/CW, mm/g	1.00 ± 0.78^b ^(n = 18)	0.91 ± 0.79^b ^(n = 89)	0.917^c^
Arrhythmia, %	55.2 (16/29)	31.7 (40/126)	0.020^a^

^a^χ^2 ^test.

^b^Mean ± SD.

^c^Mann-Whitney U test.

DT/CW = dividing diameter of tumor at diagnosis by physiological cardiac weight at diagnosis.

Finally, we acknowledge that our study may be affected by publication bias since it is based on a cumulative case series.

## Conclusions

Our literature survey reveals that younger age of the patient at the time of diagnosis and septal involvement can be regarded as significant poor prognostic factors for cardiac fibroma, a tumor that grows with physiological cardiac growth. Furthermore, our detailed statistical analyses support the following hypotheses. First, the high ratio of tumor-to-heart size may generate low cardiac output and therefore lead to a poorer prognosis. Second, the ratio of the sites of cardiac fibroma occurrence corresponds with the ratio of the muscular weight of the cardiac chamber. Third, septal involvement is a significant poor prognostic factor for cardiac fibroma regardless of tumor diameter relative to heart size, since cardiac fibroma involving the interventricular septum more frequently induces conduction system disease. However, further investigations are required to confirm these hypotheses.

## Consent

Since the data for this study were extracted and sampled from previous publications, written informed consent for publication from patients does not exist with the exception of our autopsy case. For our case, written informed consent was obtained from the patient's family for publication of this study. A copy of the written consent is available for review by the Editor-in-Chief of this journal. Furthermore, the anonymity of all patients was strictly protected.

## Competing interests

KS reports receiving research grants from Pfizer Inc., Janssen Pharmaceutical KK., Dainippon Sumitomo Pharma Co., Astellas Pharma Inc., Taiho Pharmaceutical Co., and POLA-Pharma Inc. All authors declare that they have no competing interests.

## Authors' contributions

ST and TN equally conceptualized this study, sampled publications, integrated the data, and completed the manuscript as a major contributor. MW sampled publications, carried out the histopathological evaluation, integrated the data, and revised the manuscript; YO integrated the data, revised the manuscript, and gave final approval to the manuscript as a corresponding author; TY and KK carried out statistical evaluation and revised the manuscript; TO advised the first author on cardiac fibroma as a clinical doctor; HN and MS carried out statistical evaluation; DS, TI, and KT sampled publications and extracted raw data from English and Japanese publications and integrated the data; KS confirmed histopathological and statistical evaluations and revised the manuscript as principle investigator of the work. All authors contributed to conceptualizing and writing this study. Furthermore, all authors read and approved the final manuscript.
